# Introduction to Bacterial Anhydrobiosis: A General Perspective and the Mechanisms of Desiccation-Associated Damage

**DOI:** 10.3390/microorganisms10020432

**Published:** 2022-02-12

**Authors:** Tomasz Grzyb, Aleksandra Skłodowska

**Affiliations:** Department of Geomicrobiology, Faculty of Biology, Institute of Microbiology, University of Warsaw, Miecznikowa 1, 02-096 Warsaw, Poland; grzybtom@gmail.com

**Keywords:** anhydrobiosis, desiccation, membrane lipids, nucleic acids, proteins, cellular water

## Abstract

Anhydrobiosis is the ability of selected organisms to lose almost all water and enter a state of reversible ametabolism. Such an organism dries up to a state of equilibrium with dry air. Unless special protective mechanisms exist, desiccation leads to damage, mainly to proteins, nucleic acids, and membrane lipids. A short historical outline of research on extreme dehydration of living organisms and the current state of research are presented. Terminological issues are outlined. The role of water in the cell and the mechanisms of damage occurring in the cell under the desiccation stress are briefly discussed. Particular attention was paid to damage to proteins, nucleic acids, and membrane lipids. Understanding the nature of the changes and damage associated with desiccation is essential for the study of desiccation-tolerance mechanisms and application research. Difficulties related to the definition of life and the limits of life in the scientific discussion, caused by the phenomenon of anhydrobiosis, were also indicated.

## 1. Introduction and a Short Historical Overview

Water is essential for life for all living organisms, and in a common understanding, water supports life, and lack of water results in death. At least 70% of the mass of an average cell is H_2_O [1,2]. There are many theories about the origin of life, but they all place primitive organisms in the aquatic environment. Organisms die under the influence of extreme dehydration. There are, however, exceptions to this rule. Science has known about them for at least 300 years, since Leeuwenhoek decided to dry the “animalcula” collected from the roof (these particular ones are known today as rotifers), and then rehydrate them-what resulted in the restoration of their vital functions (what he described for the first time in the letter from February 1702) [3,4]. In the second half of the 18th century, research on organisms tolerating extreme dehydration was continued by John Needham, Henry Baker, and Larazzo Spallanzani. In 1766, Spallanzani (who originally denied the existence of the phenomenon) proved that it was possible to bring back to life rotifers that had been desiccated for four years [5]. The history of research into what would later be termed anhydrobiosis has been turbulent; the phenomenon has been strongly denied (to the extent that John Needham publicly rejected his own findings, largely due to the “early” Spallanzani), disappeared from the scientific debate for decades, and finally unexpectedly became one of the key issues in nineteenth-century scientific conflicts over the theory of spontaneous generation [3].

Anhydrobiosis (gr. life without water) is predominantly described as the ability of some organisms to lose all or almost all water and enter a state of suspension where the metabolism comes to a reversible standstill or at least to a level undetectable in a laboratory [6,7]. An organism capable of anhydrobiosis tolerates extreme dehydration (desiccation), dries up to equilibrium with moderately to extremely dry air, and then restores its vital functions after rehydration [8]. According to the classic quantitative definition of Alpert [8], extreme dehydration is understood as the loss of water to a level below 0.1 g H_2_O g^−1^ dry weight (air equivalent with 50% humidity at 20 °C). This limit has caused some difficulties in recent years. It seemed that there was no continuum between desiccation tolerance and desiccation sensitivity; if an organism is sensitive to desiccation, it will die below 20% WC (water content); if it is an anhydrobiont, it will survive to less than 10% of WC [8]. In bacteria, this “no-man’s zone” is supposed to be even greater, between 30% WC (the lower limit of WC for non-anhydrobionts) and 10% of WC. The limit of 10% is to be of biological importance: it is supposed to be the limit below which it is no longer possible to form a monolayer hydration shell around proteins and lipid membranes [8,9]. The lower limit of survival of anhydrobiotic prokaryotes was determined by *Nostoc commune*, and it was 2% by mass of water [10,11].

However, while publishing the quantitative definition, Alpert noted that there were exceptions among plants [8]. As reported over a decade later by Leprince and Buitink [12], the number of described plant species surviving in the range of 10–20% WC is increasing. Unfortunately, there is no such literature data for bacteria.

Anhydrobiosis raises a number of questions that cannot be answered easily; for it deals with issues fundamental to the very nature of life. Are desiccated cells alive? They lack almost all the features of living organisms, most of all they do not show any detectable metabolic activity. However, they are not dead, because after rehydration they obviously restore their vital functions [13]. Is it a latent life as Keilin [3] defined it; or “a third state”, beyond life and death, which we would generally call cryptobiosis [13]. No wonder that Spallanzani saw it as not only a natural phenomenon but also a metaphysical one. These considerations, on the border of the philosophy of biology, go beyond the subject of this work. Nevertheless, it is worth remembering the far-reaching consequences of the phenomenon of anhydrobiosis.

Anhydrobionts can be found in all domains. In bacteria, gram-positive bacteria predominate; we know of a few desiccation-tolerant archaea. In the case of Eukaryota, among the fungi, *Saccharomyces* spp.; in plants, they are bryophytes (interestingly, probably all lichens) but rarely pteridophytes [14]. Many pollens and seeds of Angiosperms tolerate desiccation, and a peculiar phenomenon are resurrection plants, in which even leaves and roots tolerate desiccation, approx. 300 such species have been described [14]. Among animals, no vertebrate is capable of anhydrobiosis; in the case of invertebrates, desiccation tolerance is common in three types: nematodes, rotifers, and tardigrades [14]. In addition, the state of anhydrobiosis was observed in embryos of some crustaceans and insect larvae of Diptera, *Polypedilum vanderplanki* [14].

The first modern research on the mechanisms of anhydrobiosis started in the 1960s and 1970s. It was then that in the USA, researchers such as J.H. Crowe, L.M. Crowe, and J.S. Clegg, under the influence of the review by Keilin in 1959 [3], cited here, began research from a biochemical and biophysical perspective, mainly on the *Artemia* spp. [14,15] At the same time research concerning microbial (yeast) anhydrobiosis was begun in Latvian SSR (USSR), with A.I. Rapoport and M.E. Beker as the leading researchers [16,17]. A fundamental inspiration for those researchers was another historical review, the 4th revised edition of *Anabiosis* by Russian–Soviet biologist P.Y. Schmidt [17,18].

Research into anhydrobiosis has changed significantly over the last forty years. As late as 1977, the vast majority of work on anhydrobiosis focused on defense mechanisms and damage caused by desiccation [14]; the broadly understood metabolites and antioxidants were mainly studied. In the 1980s, there was a peak in sugars-related work. The breakthrough in the field of prokaryotic anhydrobiosis came in the 1990s; the publication by Potts was the first modern cross-sectional analysis of anhydrobiosis outside the world of Eukaryota.

In recent years, most of the published research has focused on cell signaling and the expression of specific genes; the “omics” revolution is a major breakthrough in the study of the phenomenon of desiccation tolerance, e.g., [12,14,19]. Most articles concern the late embryogenesis abundant proteins (LEA proteins), originally detected in plants; today their homologues are known among both microorganisms and animals [14]. A recent review, published in August 2021, focusing on the hypothetical basal metabolic processes and enzymatic activities occurring in microorganisms in an anhydrobiotic state (thereby, questioning the classical definition of anhydrobiosis, see Section 2. Terminological issues), highlights important gaps in our understanding of the qualitative and quantitative aspects of molecular and biochemical processes in desiccated cells [19].

The subject of our review is the phenomenon of anhydrobiosis among bacteria, although some processes are common to all domains. Terminological issues will be summarized and the role of water in the cells of living organisms will be presented. Next, we will discuss the structural damage that organisms face during desiccation. Understanding the nature of the processes taking place in cells during desiccation, and in particular significant structural changes, is essential for research into the mechanisms of desiccation tolerance or their application. We will pay special attention to damage to proteins, nucleic acids, and lipids.

## 2. Terminological Issues

Review of the works in the field of anhydrobiosis and tolerance to (extreme) dehydration encounters several terminological problems. Certain terms are used with different meanings depending on the field of research. Thus, before we proceed to the analysis of the issue, we will clarify the conceptual issues.

We define anhydrobiosis as the phenomenon of the ability to enter a state of reversible ametabolism or suspended metabolism due to cell desiccation. This condition is known as the state of anhydrobiosis. Consequently, organisms capable of anhydrobiotics are referred to as anhydrobionts or anhydrobiotic organisms. 

However, we should acknowledge here that some researchers tend to oppose this definition as not reflecting the complex and subtle realities of the anhydrobiosis phenomenon, while propounding the existence of basal anhydrobiotic steady-state metabolism (see [19]).

Desiccation tolerance is synonymous with anhydrobiosis in the sense that the only known mechanism for tolerating desiccation over time by initially vegetative cells is anhydrobiosis. Here, however, it should be noted that, especially among plant physiologists, there is a postulate to abandon the term anhydrobiosis in favour of desiccation tolerance (see [14]).

Desiccation is a term for extreme dehydration. This is also synonymous with the term matric water stress used by Potts [2]. Some authors distinguish between total desiccation and partial desiccation (usually identical to dehydration); in this work, partial desiccation is understood as dehydration.

It should be noted that desiccation tolerance is not the same as drought tolerance. Drought is understood as the low water content in the environment; dehydration (and in extreme cases, desiccation) is a shortage of water inside the cell. However, it is worth remembering that in vivo, these states often coexist and intertwine.

In recent years, the term xerotolerance has gained popularity in several works. It is used in various ways, as an equivalent to desiccation tolerance [20], but also as a partial dehydration tolerance, drought tolerance, or as a collective term [21]. 

## 3. The Role of Water in the Cell

To understand the importance and consequences of extreme dehydration, we must first look at the role of water in the cell. Until recently, the perception of the importance of water in biology was undoubtedly paradoxical; on the one hand, water was to be absolutely essential for life; on the other hand, its role was limited to being an inert solvent, a solvent with some unusual physicochemical properties, but still only a background for life’s molecular components [22,23,24]. The first of these claims is at least partially true; water is indeed necessary for the existence of life on Earth [23]. The second, as evidenced by the last twenty years of research, is fundamentally false [23]. The perception of passive water is still present today in graphics or diagrams; still, biomolecules, such as proteins or sugars, are placed on a “uniform black background” [22]. At the same time, one should bear in mind the pseudoscientific “interpretations” of the role of water, which gives it an almost magical role-such concepts, the so-called alternative medicine, such as water memory, structured water, and polymerized water [25].

Water conditions and participates in the molecular movements on which biochemical reactions depend. It maintains the structure and activates and modulates the dynamics of proteins and nucleic acids, it is also a means of rapid communication as a water wire [23,24]. Water acts as a reactive nucleophile, proton donor and acceptor, and mediates electrostatic interactions [23]. Water determines one of the key forces for supramolecular changes, hydrophobic interactions. It does not seem exaggerated to say after Ball that the current state of knowledge erases the dividing line between the “biological components” and their environment [22]. 

### 3.1. The Specificity of the Aquatic Environment in the Cell

The water in the cell is not the same as the bulk liquid water in an abiotic environment. Firstly, a significant part of the water in the cell is bound or at least partially immobilized and is not subject to osmotic processes [23,24]. We have to realize that the cellular environment is very “crowded” from a molecular perspective. The average distance between macromolecules in a cell is approx. 1 nm; it is only three to four layers of water molecules [23]. This generally does not meet the criteria for a bulk-like solution. 

Liquid water forms a constantly fluctuating network of hydrogen bonds, each of which has an average lifetime of approx. a picosecond [23]. Atoms in biologically active molecules can replace any bonds between each water molecule; water molecules can be bound to the surface of biomolecules with forces both stronger and weaker than the forces of interaction in bulk-like phases [24]. The exchange of places between water molecules at biological molecules takes place in the time determined in picoseconds (1–100 ps); however, these changes depend more on the local topography of the particle and exposure to competing water molecules than on their bond strength [24]. Cellular water has a rhythm that is separate from bulk water in solutions; the time of particle placement can be both longer and shorter. Although we usually actually operate in picoseconds, for example, H_2_O molecules placed in deeply concave clefts and internal cavities can be exchanged with bulk at a time determined even in microseconds [23]. 

This alteration of the companion particles makes it possible, in the first place, to change the shape of the surface of macromolecules, such as proteins; in other words, the ability of water to enter into numerous weak bonds enables the reorientation and reconfiguration of three-dimensional structures [22,24]. As noted by Ball [23], the key to success is the collaboration between structure and dynamics; and in the case of macromolecules, their dynamics cannot be decoupled from the dynamics of the solvent.

As mentioned earlier, hydrophobic interactions are a key structural driving force of solvent water. Among the many effects of these interactions, cell membranes are crucial from a biological perspective. Hydrophobic forces also influence protein folding and protein–ligand interactions [23]. We still know little about the nature of these interactions; we are not sure about the role of entropy and we do not know the exact mechanisms of these interactions; in the case of small hydrophobic particles, it is suggested that water functions as clathrates, and in the case of larger ones, capillary evaporation [23]. However, as Ball [23] notes, despite the lack of certainty about the mechanisms, we know that the dynamical collective fluctuations of interacting water molecules are as important as the chemical characteristics of hydrophobic particles (it remains an open question whether this is the nature of hydrophobic or solvophobic interactions in general).

### 3.2. The Importance of Water for the Functioning of Proteins and Nucleic Acids

Protein–water interactions determine both the structure and activity of proteins. Hydration water molecules can adopt crystallographically well-defined positions, and these can have specific functional roles [23]. In fact, the close pairing with the hydration layer particles makes water a part of the biomolecule. Thus, water can play a role in both catalytic and molecular recognition processes.

Regarding the structure of the protein, it is enough to mention that the accompanying water molecules and the hydrogen bonds formed have been shown to be of great importance for the determination of the folding funnels within energy landscapes that determine the result of the protein folding process [24]. The role of the network of hydration shells in various allosteric conformational shifts has also been described [23].

It is difficult to not have the impression that proteins use the water for whatever they can. A good example of this is the role of water molecules in enzymes. Thus, water can serve both to increase the selectivity of a substrate and to allow interaction with more substrates [23]. For example: it has been proven that different locations of water molecules in the active sites of these enzymes are at least partially responsible for the promiscuity of alkaline phosphatases [23].

What seems obvious, but at the same time is the key implication for cell desiccation, is that a certain amount of water is necessary for the biological activity of absolutely any protein [24]. Studies have shown that the minimum required for enzyme activity is to cover most of their surfaces with water molecules that form hydrogen bonds [24].

Proton transport plays an important role in the functioning of many proteins. In fact, this is one of the most common uses for in bound water in biology [23]. The extraordinary usefulness of water molecules in this area is due to the Grotthuss hopping mechanism; it leads to an anomalously fast transfer of protons in pure water [23]. The hydrogen-bonded chain of water molecules creates the so-called water wires [24]. Recent years of research show that the physicochemical basis of this process is quite complex; what is important from a biological perspective is that this transport can be both passive and actively controlled [23]. Thanks to this, it can be used by proteins; such series of water molecules linked to polar amino acids, located in the hydrophilic cavities of proteins, are a popular construct within proteins involved in proton transport [24].

In addition to being extremely important for cell membranes and proteins, water also plays an important role for nucleic acids. Both the structure of DNA and the recognition of its sequence are dependent on water molecules [24]. The DNA double helix is expanding and contracting depending on its hydration status [24]. Both the major and minor groove of DNA are hydrated by forming links with the polar atoms of the nitrogen base edges; the orientation of water molecules thus depends on the bases and their sequence [24].

## 4. Desiccation-Associated Damage

**Figure 1:**
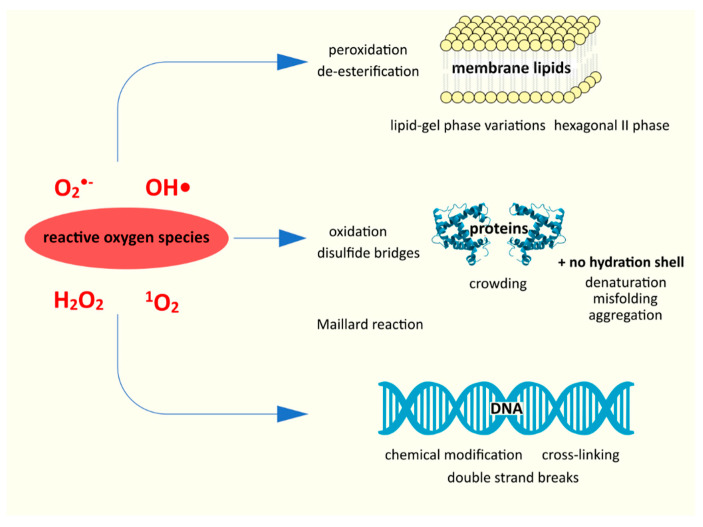
The main mechanisms of damage associated with desiccation (data from [20]; “Fluid mosaic model of cell membrane” by Jerome Walker; under Walker. license, it was adapted and used).

Having briefly discussed the role of water, we can look at the damage caused to bacteria by desiccation and its mechanisms (Figure 1). At the very beginning, however, we need to explain the difference between desiccation and osmotic stress; we need to understand how little water remains during extreme dehydration. The immediate environment of a cell affected by desiccation is air, and that of a cell affected by osmotic stress, an aqueous solution [26,27]. Even stress experienced by extreme halophiles (such as archaea of the genus *Halobacterium*) is characterized by much less water loss than the matric water stress (another term for desiccation stress) experienced by anhydrobionts, during which even a single layer of the hydration shell around the macromolecules is missing [10,26]. In general, it is assumed that organisms sensitive to desiccation are not able to survive a decrease in the water content in a cell below 0.3 g H_2_O g^−1^ dry weight; anhydrobionts withstand contents below 0.1 g H_2_O g^−1^ dry weight [10]. The bacterial responses to osmotic stress, however, may be synonymous with the first phase of slow desiccation [26]. It should also be borne in mind that environmental conditions are significantly different from those in the laboratory-in natural environments, various stress factors overlap and it is often difficult to separate them. An example of an overlapping osmotic and desiccation stress would be intertidal microbial mats that are periodically submerged by seawater and then, for many days of the year, desiccated and salted [2].

Before we proceed into a detailed critical discussion of the damage, it is important to look at the damage to a cell from a broader perspective. We know that in extreme dehydration, two stages can be distinguished. In the initial phase of the desiccation process, extracellular water is removed. This leads to an increase in the concentration of substances in the external environment and results in osmotic stress [20,28]. When the environment of the bacteria is solid, the cell–air contact increases. This is when the actual extreme dehydration occurs, including the loss of hydration shells of macromolecules in the cell [20].

From the very beginning of the process, water loss is associated with certain specific mechanical and structural changes; there is a decrease in turgor, contraction of the cell; the cell itself becomes even more “crowded” [20,21]. The increase in the concentration of various types of ions and metabolites affects the cycles of biochemical reactions; in addition, the reduced space limits the mobility of molecules, which further affects the metabolic processes [20,28]. Desiccation results in abnormally large amounts of reactive oxygen species (ROS) in aerobic bacteria, such as hydrogen peroxide H_2_O_2_, superoxide anion O_2_^•−^, or the hydroxyl radical •OH [20,29]. This is probably due to two issues; first, during desiccation, individual enzyme proteins as well as the electron transport chain as a whole are damaged [28,29]. This prevents the cell from neutralizing the ROS. This theory is confirmed by studies showing a positive correlation between the reduction in the respiration rate and the survival rate of dehydration [28]. A 10-fold increase in oxidative processes due to dehydration was measured in yeasts [30]. As indicated by França et al. [29], changes related to desiccation generally inhibit antioxidant protection systems. In turn, the mechanical consequences of desiccation, such as cell shrinkage, an increase in intracellular concentration, a decrease in cytosol fluidity, even promote the accumulation of ROS [29].

The second cause of ROS overproduction may be increased by direct exposure to air during and after extracellular water depletion, which may also explain the appearance of exogenous ROS, especially in the presence of photosensitizers [28]. Exposure to solar radiation also affects ROS production [20,29]. Moreover, as pointed out by Ogilby [31], e.g., singlet oxygen formed in the air environment is more reactive (due to, inter alia, its longer existence). The formation of the reactive oxygen species is also driven by the Fenton reaction (Fe^2+^ + H_2_O_2_ → Fe^3+^ + OH^−^ + •OH) or, more broadly, the Haber-Weis (net equation: O_2_^•−^ + H_2_O_2_ → O_2_ + OH^−^ + •OH), of which the Fenton reaction is a part (for both reactions, see [32]). The damage to the transport proteins leads to the accumulation of Fe^2+^ ions; iron ions catalyze the Fenton reaction, resulting in even greater production of the hydroxyl radical [21].

There is now consensus that oxidative stress is key to understanding the desiccation process; ROS are one of the major, if not major, drivers of negative change [28,30,33]. Reactive oxygen species damage both proteins (denaturation) and nucleic acids and lipids (peroxidation and de-esterification) [29]. Apart from these, physicochemical changes of aggregate phases, Maillard reactions, and others also play an important role [21]. 

### 4.1. Damage to Proteins

Protein dehydration itself can lead to their denaturation, which results in the loss of biological functions, total enzymatic, synthetic, transport, repair, upon rehydration [21,29,34]. The reduction in or loss of the hydration shell leads to interactions with molecules with which proteins are not normally in contact; this also leads to denaturation and aggregation. This phenomenon is exacerbated by the increased concentration of ions in the cell [28]. The lack of water as an environment creating hydrophobic interactions leads to the exposure of hydrophobic regions of proteins, so far located in the core of these particles. These regions increase the susceptibility to aggregation that occurs [20]. In addition to the loss of their primary functions (and, therefore, the occurrence of metabolic stress), proteins in the form of aggregates cause damage; they induce the formation of ROS, lipid peroxidation, and rearrangement of cell membranes [20,35]. Conformational changes related to dehydration block antioxidant enzymes and damage the electron transport chain-this enhances the reaction cascade by accumulating ROS [10,21]. The malfunction of transport proteins leads to the accumulation of Fe^2+^ ions mentioned in the previous subsection, catalyzing the Fenton reaction [21].

Proteins in the dehydrated state of the cell also become an easy substrate of the Maillard reaction (which is a non-enzymatic browning reaction), stimulated by reactive oxygen species. In the Maillard reaction, covalent bonds between the carbonyl group of saccharides and the primary amines of nucleic acids or amino groups of proteins are formed [21,36]. The result is irreversible polymerization, the formation of various aggregates, destroying both proteins and DNA [20,36]. In addition to the Maillard reaction, the proteins themselves are oxidized. Sulfur-containing amino acid substituents and aromatic groups are particularly vulnerable to ROS attack; their oxidation leads to a number of modifications of protein structures, e.g., to the formation of disulfide bridges [28]. Proteins subjected to such oxidation are usually more prone to proteolysis and lose their biological functions [29,30]. The opposite is the case with carbonylated proteins; they become resistant to degradation and accumulate in bacterial cells during desiccation [2,26,29]. In oxidized proteins, uncontrolled oxidation of thiol groups to sulfonic acid residues can also take place [29].

### 4.2. Damage to Nucleic Acids 

Maintaining the genetic information carried by DNA is absolutely essential for a cell to survive and function after potential rehydration. The key factors here are DNA stability during cell dehydration and the ability to repair DNA after rehydration [29]. DNA should be fully hydrated, as changes in the hydration pattern can disrupt replication and transcription [30].

Many desiccation-induced DNA defects are mediated by covalent modifications, such as the Maillard reaction, DNA cross-linking, and double-strand brakes [20]. As reported by Greffe and Michiels [20], there is still no comprehensive overview of DNA damage related to oxidative stress. We know that there are chemical modifications such as alkylation and oxidation, depurination, and crosslinking. One of the effects of oxidation is the degradation of pyrimidines into hydantoin rings. The resulting rings prevent replication, obstructing DNA polymerases. At the same time, during desiccation, proteins are damaged, including those that are involved in the protection or repair of DNA, such as Dps, H-NS, or RecA proteins [21]. Thus, during desiccation, DNA accumulates damage, including what will lead to cell death. Extreme dehydration affects the genetic information of a cell in two ways; it leads to simultaneous damage to the DNA structure and destruction/inactivation of protective and/or repair mechanisms [20,21].

### 4.3. Membrane Lipids

#### 4.3.1. Lipid Characteristics of the Cytoplasmic Membrane

To fully understand the effects of cell desiccation on lipid membranes, we need to summarize the current understanding of these. The classic image of biological membranes is the fluid mosaic model proposed by Singer and Nicholson in 1972 [37,38]. It assumes the existence of a lipid bilayer within which proteins are located in various ways. In this model, membrane lipids are in the liquid crystalline state and are laterally evenly distributed to form a homogeneous structure [38,39,40]. At the same time, however, it was known that cytoplasmic membranes must be laterally polarized in order to create a specific environment for some membrane proteins [41]. The Singer and Nicholson model has been subject to revisions over the years. Thus, in vitro studies with model lipids and studies with eukaryotic cells revealed the laterally heterogeneous nature of lipid membranes [42]. These local structures are referred to as lipid domains. According to Strahl and Errington [42], from the conducted research, three main factors determining the formation of lipid domains can be identified. The first is the chemical structure of the lipid head groups and the associated charge, and the physical shape of the lipid species; the second is the lipid fatty acid moieties and the associated differences in the fluidity and packing of the lipid bilayers [42]. The third major factor is the phase behaviour of lipid bilayers [42].

However, it was still assumed that lipid domains were a characteristic for Eukaryota [41]. This state-of-the-art was analogous to the development of anhydrobiotic studies, regarding microbial anhydrobiosis, it has been focused mainly on yeast (one must remember, though, that this situation is highly influenced by historic and commercial importance of *Saccharomyces cerevisiae*), cf. [5,16,28,29,43,44,45,46,47,48,49,50,51]. Regarding lipids and lipid domains, it is known, for example, that dehydration in yeast cells causes the decrease in spacing between membrane phospholipids and ordering of the hydrocarbon chains and additionally may disorder the hydrophobic chains of the lipid, as a result of the division of amphiphilic molecules between the aqueous cytoplasm and the lipid phase of membranes during drying of [44].

However, through a series of studies, supported by spectroscopic methods as well as microscopic visualizations, the lateral heterogeneity of the cytoplasmic membrane in bacteria was demonstrated [41,42]. 

**Figure 2:**
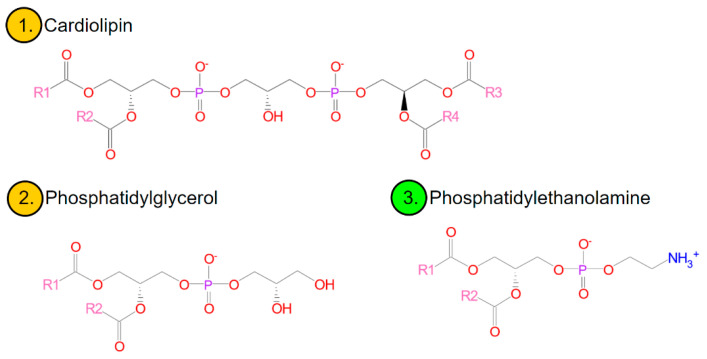
Major bacterial phospholipids. Orange rim-anionic lipids and green rim-ionically zwitterionic (data from [52,54]).

Membrane lipids in bacteria are mainly three glycerophospholipids, ionically, zwitterionic phosphatidylethanolamine (PE) and anionic, phosphatidylglycerol (PG) and diphosphatidylglycerol (known as cardiolipin-CL) [52,53] (Figure 2). In smaller amounts, although commonly, the membranes contain anionic phosphatidic acid, glucolipids, or positively charged lysine-phosphatidylglycerol (lysyl-PG) [42]. The existence of regions particularly rich in cardiolipin has been shown in many species of bacteria, including, e.g., *Escherichia coli*, *Bacillus subtilis*, *Pseudomonas putida*, *Enterococcus faecalis*, and *Streptococcus pyogenes*. Polar and negatively charged CL domains affect the activity and localization of proteins (e.g., polar or at the division site) [42,53]. A similar role can be played by anionic PG [52,53] or domains characterized by a locally increased membrane fluidity [42]. Anionic phospholipids also favour the formation of structures other than bilayers (which will be discussed later)-which may be important in the course of cell division [41].

**Figure 3:**
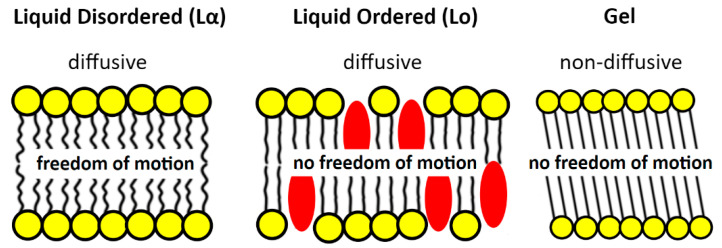
Structure and differences between the phases L_α_ (disordered liquid crystal), the intermediate form L_o_ (liquid-ordered), and L_β_ (gel); red oval–hopanoids (data from [38,55]; elements of the figure by Faller [38] were used).

Apart from the anionic domains and those conditioned by the increased fluidity of the membrane, there are also so-called lipid rafts. These regions have been extensively studied in Eukaryota, but their existence in bacteria has long been denied. The formation of membrane rafts results from a change in the phase of matter-from a lamellar disordered liquid-crystalline phase (L_α_; liquid-crystalline phase, liquid-disordered phase) to an intermediate liquid-ordered phase (L_o_) [38,42,53] (Figure 3). These domains play an important role in cellular processes, such as signal transduction and membrane transport [42]. Phase transitions L_α_ → L_o_ are induced by cholesterol, which, as a rule, is not present in bacteria. However, as shown by recent research, in bacteria sterols in this role can be replaced by hopanoids [42,55,56].

Lipid rafts are just one example of the occurrence and role of the various lipid phases in the functioning of bacterial membranes. Changes in phases and structures are also the main point of damage to lipid membranes during dehydration and desiccation; so, in the next section the current state of knowledge on this subject will be briefly summarized.

#### 4.3.2. Phases and Phase Transitions of Lipids

**Figure 4:**
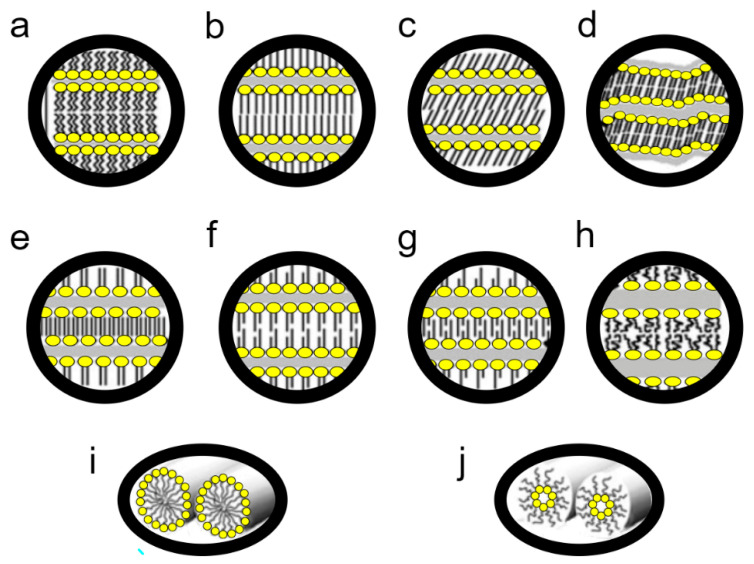
Selected lipid phases. (1) lamellar: (**a**) crystalline, Lc; (**b**,**c**) gel L_β_ differing in the tilt of the chain types; (**d**) rippled gel P_β_; (**e**–**g**) gel L_β_ with a varying interdigitation; (**h**) liquid crystal, Lα. (2) hexagonal: (**i**)-regular, H_I_; (**j**)-inverted, H_II_ (data from [57], figure according to Koynova and Tenchov [57], adapted and redrawn).

The basic state of the matter of biological membranes is the liquid-crystalline lamellar (L_α_) phase. Membrane lipids, however, can pass into a number of other phases (Figure 4). If we use the thermotropic perspective to describe the possible forms, the “lowest” crystalline-subgel (L_c_) phase occurs. As the temperature increases, it transforms into the gel lamellar phase (L_β_), a process called subtransition. As the temperature increases, L_β_ melts and transforms into the L_α_ phase, i.e., the liquid crystalline phase dominating in lipids [40]. This phase transition is known as the main phase transition.

L_α_ under the influence of temperature increase undergoes mesomorphic transitions into non-lamellar liquid crystalline phases: hexagonal (H), micellar (M), and cubic (Q) [40,57]. The most common transition of this type is the transformation of L_α_ to H_II_—the inverted hexagonal phase (Figure 4). Moreover, mesomorphic transitions also occur at a constant temperature, when the water content changes; a decrease in water content causes L_α_ transitions into inverted non-lamellar structures, growth into normal non-lamellar structures [57].

There are also numerous modifications, e.g., the gel phase may have various modifications. So, we have the gel phase interdigitated, partially interdigitated, noninterdigitated; tilted chains or not. It happens that before the transformation of L_β_ → L_α_, the so-called pretransition, the L_β_ phase first transforms into P_β_, the rippled gel phase (Figure 4) [57,58]. There are also intermediate structures, such as the aforementioned L_o_, an ordered liquid lamellar phase that determines the formation of lipid rafts (5).

The functioning of bacteria is based on maintaining a component balance between lipids forming bilayers and those that favour other mesoforms; the length of chains, the number of unsaturated bonds in acyl chains, and the composition of hydrophilic heads are important [39,59]. There is constant balancing on the line between the main phase transition, the intermediate L_o_ form, and the transition to inverted non-laminar phases. At the same time, local non-lamellar structures are desirable and play important roles, e.g., in fusion and separation of membranes [39,59]. The local presence of both H_II_ and L_β_ phases modulates the activity of membrane proteins [40]. It is this dynamic equilibrium that is completely destabilized during desiccation.

#### 4.3.3. Damage to Cell Membrane Associated with Desiccation

Before the lipid phase balance is destabilized, mechanical stress affects the cytoplasmic membrane. During dehydration, the cell volume decreases, while the membrane surface remains unchanged, because it exhibits low lateral compressibility [28]. Changing the surface area to volume ratio leads to deformation of the plasma membrane [20]. It should also be noted that the cytoplasmic membrane, apart from lipids, also consists of proteins-and these are subject to the damage described earlier.

During desiccation, the packing density of the phospholipid heads increases, as there are no water molecules surrounding them. This, in turn, leads to increased van der Waals interactions between the chains of fatty acid residues, which results in an increase in the value of T_m_, i.e., the temperature of the main phase transition [21,26,29]. This signifies the transition of the membrane from the L_α_ liquid crystal phase to the L_β_ gel phase. However, the membrane fragments and the different types of lipids have different T_m_ values; phase transitions take place heterogeneously and at different times. This results in the separation of individual components of the cytoplasmic membrane, which may lead to its permanent disruption. Analogously, during rehydration, there will be a phase transition in the opposite direction; all these changes lead to membrane leakage upon rehydration [21]. Further, extreme dehydration leads in turn to the mesoformic transition of L_α_ liquid crystal structures to the inverted hexagonal H_II_ state (the transition temperature is lowered) [20,21,60]. When the interphase balance is lost, another phase separation takes place, which may result in membrane fusion and internalization [20,28]. Uncontrolled phase changes also lead to impaired membrane function, described in the previous section, and protein aggregation. 

Membrane dehydration leads to the formation of reactive oxygen species, as the continuity of the respiratory chain is broken, the superoxide radical anion •O_2_^−^ is accumulated, and the ionic balance and pH balance are disturbed [21]. During desiccation, the cytoplasmic membrane itself becomes particularly susceptible to oxidative stress. Reactive oxygen species lead to peroxidation and de-esterification of lipids, especially in the middle stage of the process of extreme dehydration [20,29,30].

## 5. Summary

What is special and beautiful about anhydrobiosis is the undermining of our dogmas. As noted by Leprince and Buitink [14], the growing understanding of the phenomenon of desiccation tolerance will challenge the biological dogmas of water, life, and evolution. For, as Chaplin [24] asks in his article, do we not “minimize” the importance of water in cell biology? The state of anhydrobiosis, which is mainly described as a state of ametabolism, but a reversible ametabolism, challenges the definitions and descriptions of life and death. Anhydrobiosis, called “a peculiar state of biological organization” by Clegg [13], forces us to rethink our understanding of cellular life. At the same time, anhydrobionts cause us to raise a question about the limits of the phenomenon of life, as they are found in the most hostile environments on Earth, often being polyextremophiles, surviving in simulations of Martian or outer space conditions.

We discussed the nature and mechanisms of structural damage related to the desiccation of bacterial cells (metabolic and physiological issues related to gene expression were discussed in [19]). We presented the issue of the importance of water in the cell, discussed the often confusing terminological issues, outlined the historical perspective and the state of modern research related to desiccation tolerance in bacteria. In short, we discussed the elements, the ordering of which seems to be necessary for the work on the mechanisms of desiccation tolerance in bacteria, their applications, and ecology.

We are aware that, contrary to the predictions from years ago, anhydrobiosis is not a simple process, it is not a problem to be solved quickly [61,62,63]. Particularly important gaps in our understanding of anhydrobiosis concern the processes taking place in desiccated cells [19]. Nevertheless, the leap forward in “omics” techniques enables us to better understand life in a state of extreme dehydration. Leprince and Buitink [14] look for hope for increasing our knowledge in functional genomics, systems biology, and comparative research. Increasingly, we already know what probably works for desiccation tolerance, although we still too rarely know how it works. Therefore, it is important to look at the mechanisms of cellular damage faced by anhydrobionts. The very discovery of the mechanisms of anhydrobiosis, like any discovery of the mechanisms of extremophiles, opens new doors for biological sciences [48].
